# Unusual Bilateral Paramolars Associated with Clinical Complications

**DOI:** 10.1155/2015/851765

**Published:** 2015-05-21

**Authors:** A. N. Sulabha, C. Sameer

**Affiliations:** ^1^Department of Oral Medicine and Radiology, Al-Ameen Dental College and Hospital, Bijapur, Karnataka 586108, India; ^2^Department of Oral and Maxillofacial Surgery, Al-Ameen Dental College and Hospital, Bijapur, Karnataka 586108, India

## Abstract

Paramolars are rare supernumerary structures of maxillofacial complex that occur buccally or lingually near the molar row. Predominantly these occur singly; bilateral presentation is very rare. This paper reports two unusual bilateral presentations of paramolars with clinical complication and its management. One of the cases in the present paper also documents the cooccurrence of bilateral paramolars and microdontia of single tooth and one of its paramolars presented with multilobed crown with an anomalous buccal tubercle.

## 1. Introduction

Supernumerary teeth are the teeth or tooth substances in excess of normal dental formula of deciduous or permanent dentition [1–3]. Supernumerary teeth have marked predilection for maxilla over mandible and are most commonly found in incisal region followed by molar region [4, 5]. Supernumerary teeth may occur singly in about 76–86%, double in about 12–36%, and multiply in about 1% of the cases [2].

Supernumerary structures occurring in molar region can be divided into paramolars and distomolars [3]. Paramolar is usually small and dysmorphic supernumerary tooth located buccally or palatally/lingually to one of the molar series. Distomolars are located distal or distolingual to third molars [3]. Literature has few countable reports of paramolars as these are less often seen. Predominantly these occur singly and very rarely is bilateral occurrence seen [5]. Hereby we report two unusual cases of bilateral paramolars in maxilla associated with clinical complications. Cooccurrence of the bilateral paramolar and microdontia of single tooth which is rare clinical presentation was noted in one of our cases.

## 2. Case-1

**Figure 1:**
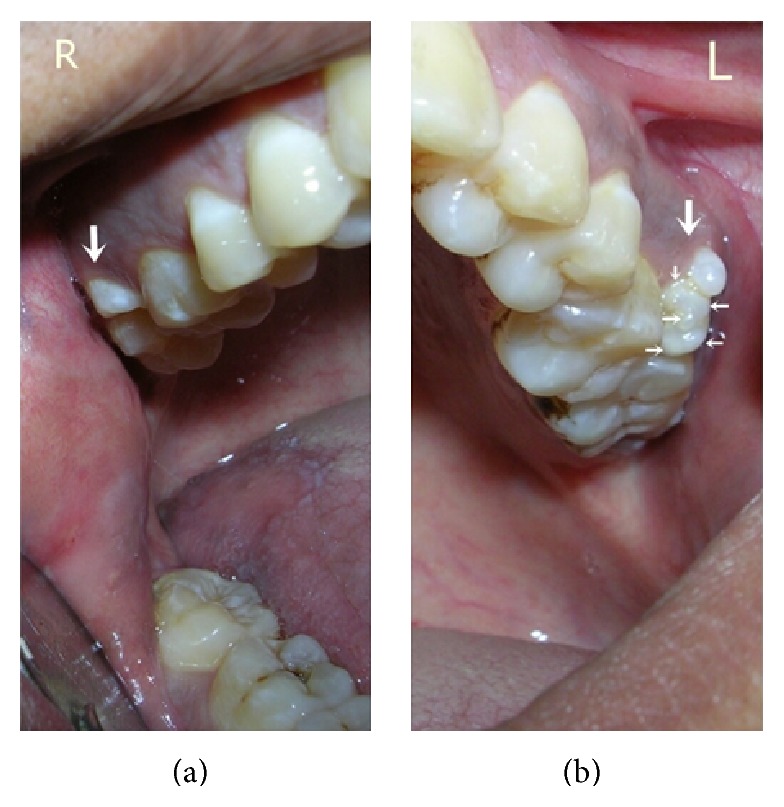
Showing the bilateral paramolars on right and left (multilobed) sides in case-1.

A 35-year-old female patient reported to the Department of Oral Medicine and Radiology with chief complaint of food lodgment in left upper back region. Intraoral examination revealed bilateral paramolars in maxilla located between first and second molars on buccal aspect. The paramolar on right side was vertically oriented having molariform crown morphology. Paramolar on the left side was vertically oriented with multilobed crown. The paramolar had five small lobes separated by noncarious grooves with anomalous tubercle on its buccal aspect (Figure 1). Surrounding soft tissue was inflamed. Microdontia was noted with left maxillary third molar which presented with very small heart shaped crown. Patient's medical and family histories were noncontributory and patient did not show any sign of syndromic features.

**Figure 2:**
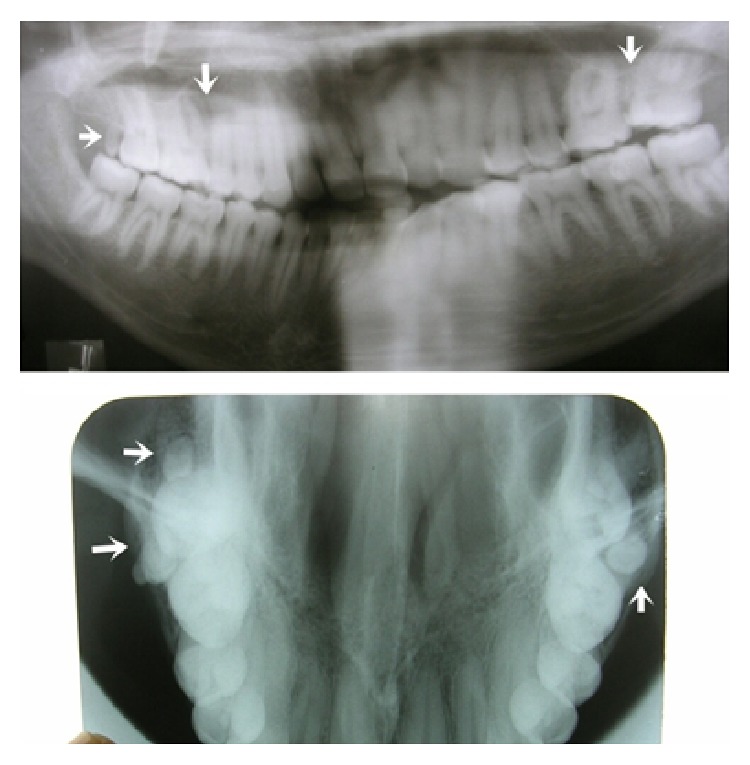
Showing the bilateral paramolars in occlusal and panoramic view in case-1.

**Figure 3:**
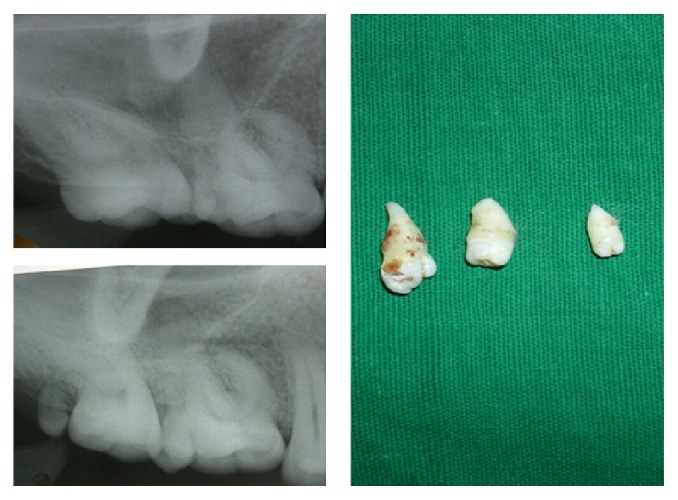
IOPA showing the paramolars and microdontic third molar and the surgical specimen of case-1.

Radiographic examination by intraoral periapical radiograph in occlusal and panoramic views showed multilobed paramolar with anomalous tubercle on buccal aspect with conical root on left side, microdontia with left third molar involving both crown and root, and paramolar with incomplete root on right side. Horizontal bone loss was noted between left paramolar and second molar (Figure 2). Treatment modalities consisted of extraction of supernumeraries along with microdontic third molar as these areas were inaccessible to maintain oral hygiene and patient had already had developed localized periodontitis in adjacent area of left paramolar region. Extracted multilobed paramolar showed caries in its proximal aspect along with anomalous tubercle arising from the cementoenamel junction extending till half the crown length (Figure 3).

## 3. Case-2

**Figure 4:**
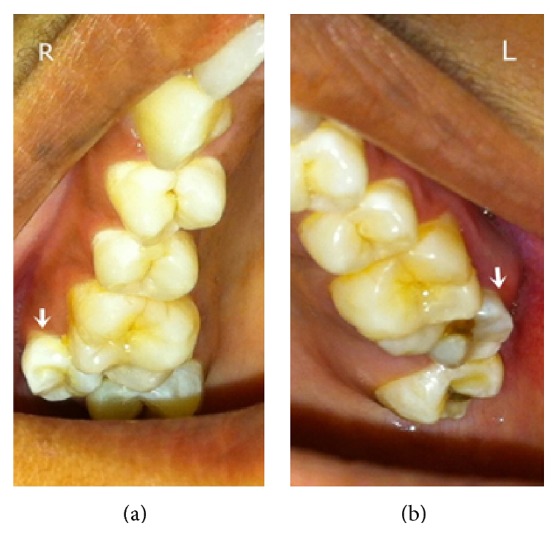
Showing the bilateral paramolars in case-2.

A 25-year-old female patient reported to Department of Oral Medicine and Radiology with complaint of decay in tooth in upper left back region. Intraoral examination revealed bilateral presentation of paramolars in maxilla between first and second molars on buccal aspect. Examination of right paramolar revealed vertically oriented tooth with small molariform crown. Left side paramolar was also vertically oriented with caries involving the mesioproximal aspect (Figure 4) and the tooth was tender on percussion. Surrounding soft tissue was inflamed. Patient's medical and family histories were not significant and she did not have syndromic features.

**Figure 5:**
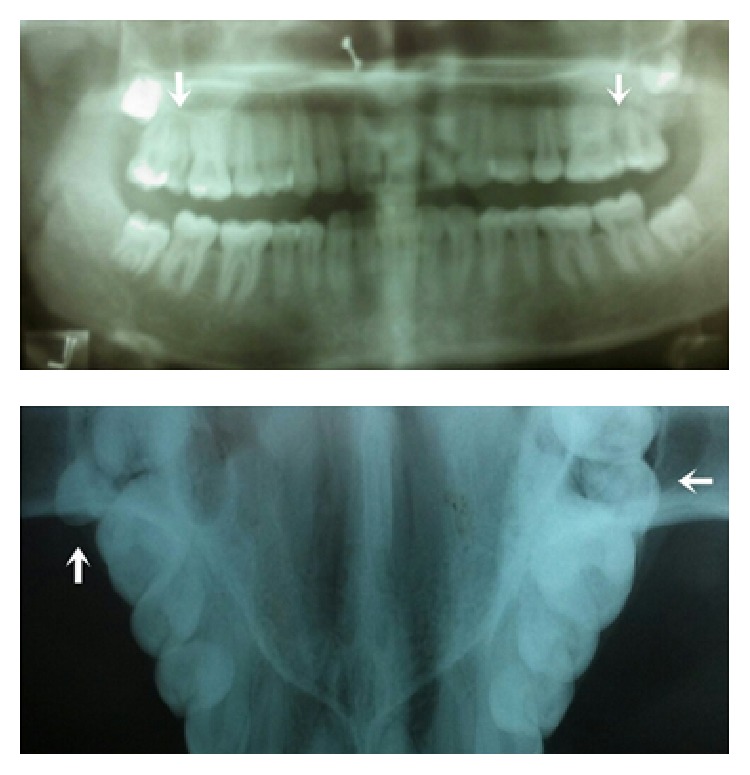
Showing bilateral paramolars in occlusal and panoramic views in case-2.

**Figure 6:**
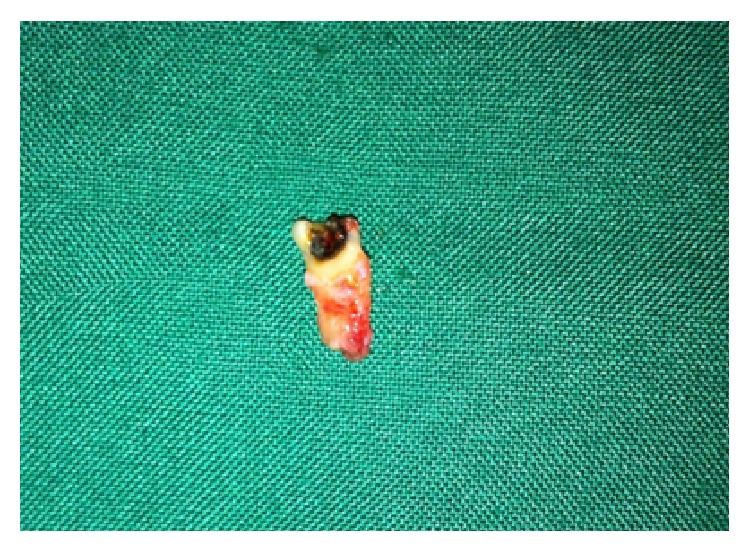
Showing extracted paramolar of case-2.

Radiographic examination by occlusal and panoramic view showed horizontal bone loss between left paramolar and first molar with caries involving the pulp of paramolar (Figure 5). Management approach consisted of extraction of the left paramolar as caries was involving the pulp of paramolar and it was compromising the adjacent periodontal health. The other paramolar was asymptomatic and it was kept under observation as patient was not willing for its treatment. Extracted left paramolar showed caries on its proximal surface with incomplete root formation (Figure 6).

## 4. Discussion

Supernumerary teeth are defined as those which are an addition to the normal series of deciduous or permanent dentition [3]. Classification of supernumerary teeth according to chronology can be predeciduous, past permanent, or complementary. According to morphology it can be odontoma, supplemental or eumorphic and rudimentary or dysmorphic having abnormal shape and small size, and dysmorphic morphology includes the conical, tuberculate, and molariform types. According to topography, it can be mesiodens, paramolars, distomolars, and parapremolar and according to orientation it can be vertical, inverted, and transverse [3, 5].

Paramolars are relatively uncommon supernumerary anomalies occurring in molars series with prevalence of 0.09–0.29% [2]. These are most commonly located between second and third molars on buccal aspect or lingual aspect and are rarely seen between first and second molars [6]. Supernumerary teeth have marked predilection for males [2]. In the present paper both the cases were seen in female patients and paramolars occurred bilaterally on buccal aspect between the first and second molars.

Exact mechanism of their development is still unclear but various factors such as genetic and environmental factors have been proposed. According to dichotomy theory supernumerary teeth such as paramolar arise from third tooth bud arising from dental lamina near permanent tooth bud or possibly by splitting of permanent bud itself. According to theory of phylogenetic reversion, paramolars may be an atavistic appearance of fourth molar of primitive dentition. Hyperactivity theory is the most acceptable one. It states that supernumerary teeth such as paramolars are result of local, independent conditional hyperactivity of dental lamina. According to this lingual extension of additional tooth, bud gives supplemental or eumorphic tooth. Rudimentary form arises from proliferation of epithelial remnants of dental lamina induced by presence of complete dentition [2, 5].

Microdontia is a rare phenomenon. It is term used when teeth are smaller than normal (outside the limits of variation) [7]. Microdontia can be generalized or relatively generalized or may affect single tooth. Generally lateral incisor and third molars are involved commonly when it affects single tooth. Microdontia of single tooth may be further classified as microdontia of whole tooth, microdontia of only crown, and microdontia of root alone. Both genetic and environmental factors play role in formation of microdontia [7, 8]. In the present paper, case-1 showed microdontia with single tooth involving both crown and roots of left maxillary third molar. Their cooccurrence with bilateral paramolars may be coincidental.

Often supernumerary teeth are associated with syndromes like cleidocranial dysplasia, Gardener's syndrome, Fabry-Anderson syndrome, Ellis-van Creveld syndrome, cleft lip and palate, and so forth [2]. Microdontia is also often associated with syndromes like Gorlin-Chaudhry-Moss syndrome, William's syndrome, Hallermann-Streiff syndrome, orofaciodigital syndrome, and so forth [8]. Both of our patients did not show any syndromic manifestations.

Extensive review of international English literature revealed only 10 reports of bilateral paramolars in maxilla and mandible [2, 4, 6, 9–13]. Parolia and Kundabala [3] reported bilateral maxillary paramolars with caries in adjacent molar tooth. Hou et al. [13] presented bilateral paramolars in maxilla with localized periodontitis. Omal et al. [4] reported bilateral paramolars with distomolars. Bargale and Kiran [8] reported a case of supernumerary teeth in mandible with generalized microdontia. In contrast to all these cases, case-1 in the present paper showed concomitant occurrence of bilateral paramolars with microdontia of third molar. Multilobed paramolar with anomalous tubercle on its buccal aspect was noted which is very rare and till date has not been documented. To the best of the authors' knowledge, such clinical scenario involving the dental anomalies of bilateral paramolars with unusual shape associated with clinical complications and microdontia has not been reported.

In differential diagnosis of supernumerary structures in molars fused supernumerary and paramolar tubercle has to be considered. Paramolar tubercle is anomalous or stylar cusp or supernumerary inclusion or eminence occurring on buccal surface of both upper and lower premolars and molars [5]. In the present paper, left paramolar of first case presented with anomalous tubercle on its buccal aspect.

Numerous complications can occur due to supernumerary teeth such as crowding, malocclusion, delayed eruptions, localized periodontitis, spacing, caries, root resorption, dentigerous cyst, and abnormal root development [2, 3]. Both of the cases presented with clinical complication of caries with paramolars and localized periodontitis in adjacent areas.

Radiographs play a vital role in detecting the supernumerary teeth as most often paramolars are impacted. Accurate radiographic localization can be done by parallax method as paramolars often overlap with the molars. CBCT and spiral computed tomography also aid in making the confirmatory diagnosis [5]. Clinical management of paramolars depends on position of paramolar and its potential effect on the adjacent structures. Extraction or observations are the treatment modalities [3]. In the present cases extraction of both paramolars was carried out to facilitate proper oral hygiene in case-1, and in case-2 extraction of decayed paramolar was carried out.

To conclude, paramolars are unusual entities which predominantly occur singly and rarely bilaterally and may be associated with complications. Clinician should be aware of dental anomalies involving the numbers, size, and shape and should manage them appropriately to minimize the complications.

## References

[1] Fuentes R., Borie E., Beltrán V. (2012). Radiographical and macroscopical visualization of fourth molars: a report of four cases in maxilla. *International Journal of Morphology*.

[2] Nayak G., Shetty S., Inderpreet S., Pitalia D. (2012). Paramolars—a supernumerary molar: a case report and over view. *Dental Research Journal*.

[3] Parolia A., Kundabala M. (2010). Bilateral Maxillary paramolars and endodontic therapy: a rare case report. *Journal of Dentistry Tehran University Medical Sciences*.

[4] Omal P. M., Jacob V., Lonapan J., Kurian A. (2011). Bilateral fourth molars with paramolars in maxilla. *Kerala Dental Journal*.

[5] Nagaveni N. B., Umashankar K. V., Radhika N. B., Reddy P. B., Manjunath S. (2010). Maxillary paramolars report of a case and literature review. *Archives of Orofacial Sciences*.

[6] Shetty Y. N. (2012). A rare case of bilateral maxillary paramolars between 1st and 2nd molars. *Journal of Orofacial Research*.

[7] Sharma A. (2001). Unusual localized microdontia. *Journal of Indian Society of Pedodontics and Preventive Dentistry*.

[8] Bargale S. D., Kiran S. D. P. (2011). Non-syndromic occurrence of true generalized microdontia with mandibular mesiodens—a rare case. *Head and Face Medicine*.

[9] Kariya P. B., Mallikarjun R., Singh S. (2014). Rare combination of paramolar and distomolars supernumerary teeth in a 15 year old male adolescent. *BMJ Case Reports*.

[10] Dhull K. S., Acharya S., Ray P., Yadav S., Prabhakaran S. D. (2012). Bilateral maxillary paramolars: a case report. *Journal of Dentistry for Children*.

[11] Dhull K. S., Dhull R. S., Panda S., Acharya S., Yadav S., Mohanty G. (2014). Bilateral mandibular paramolars. *International Journal of Clinical Pediatric Dentistry*.

[12] Mayfield A., Casamassimo P. S. (1990). Bilateral paramolars and fourth molars. *Oral Surgery, Oral Medicine, Oral Pathology*.

[13] Hou G. L., Lin C. C., Tsai C. C. (1995). Ectopic supernumerary teeth as a predisposing cause in localized periodontitis. Case report. *Australian Dental Journal*.

